# Use of low-value pediatric services in the Military Health System

**DOI:** 10.1186/s12913-020-05640-5

**Published:** 2020-08-20

**Authors:** Tracey Pérez Koehlmoos, Cathaleen Madsen, Amanda Banaag, Qiong Li, Andrew J. Schoenfeld, Joel S. Weissman

**Affiliations:** 1 Uniformed Services University of the Health Sciences, 4301 Jones Bridge Road, Bethesda, MD 20814 USA; 2 Henry M. Jackson Foundation for the Advancement of Military Medicine, 6720A Rockledge Drive, Bethesda, MD 20817 USA; 3 Center for Surgery and Public Health, Harvard Medical School, 1620 Tremont Street, Boston, MA 02120 USA

**Keywords:** Pediatrics, Low-value care, Child health, Military health system, Big data, TRICARE

## Abstract

**Background:**

Low-value care (LVC) is understudied in pediatric populations and in the Military Health System (MHS). This cross-sectional study applies previously developed measures of pediatric LVC diagnostic tests, procedures, and treatments to children receiving care within the direct and purchased care environments of the MHS.

**Methods:**

We queried the MHS Data Repository (MDR) to identify children (*n* = 1,111,534) who received one or more of 20 previously described types of LVC in fiscal year 2015. We calculated the proportion of eligible children and all children who received the service at least once during fiscal year 2015. Among children eligible for each measure, we used logistic regressions to calculate the adjusted odds ratios (AOR) for receiving LVC at least once during fiscal year 2015 in direct versus purchased care.

**Results:**

All 20 measures of pediatric LVC were found in the MDR. Of the 1,111,534 eligible children identified, 15.41% received at least one LVC service, and the two most common procedures were cough and cold medications in children under 6 years and acid blockers for infants with uncomplicated gastroesophageal reflux. Eighteen of the 20 measures of pediatric LVC were eligible for comparison across care environments: 6 were significantly more likely to be delivered in direct care and 10 were significantly more likely to be delivered in purchased care. The greatest differences between direct and purchased care were seen in respiratory syncytial virus testing in children with bronchiolitis (AOR = 21.01, 95% CI = 12.23–36.10) and blood tests in children with simple febrile seizure (AOR = 24.44, 95% CI = 5.49–108.82). A notably greater difference of inappropriate antibiotic prescribing was seen in purchased versus direct care.

**Conclusions:**

Significant differences existed between provision of LVC services in direct and purchased care, unlike previous studies showing little difference between publicly and privately insured children. In fiscal year 2015, 1 in 7 children received one of 20 types of LVC. These proportions are higher than prior estimates from privately and publicly insured children, suggesting the particular need to focus on decreasing wasteful care in the MHS. Collectively, these studies demonstrate the high prevalence of LVC in children and the necessity of reducing potentially harmful care in this vulnerable population.

## Background

Healthcare costs in the United States have steadily increased over the last 20 years [1], reaching an estimated $2.9 trillion in spending in 2014 [2]. While this accounts for nearly $10,000 per person per year in 2017 [3], this expenditure does not necessarily translate into better health outcomes. Between 10 and 14% of all-payer healthcare spending represents inefficiencies in care delivery and overtreatment [4]. At best, these interventions provide no benefit to the patient, and at worst, they potentiate harm. Such care is broadly defined as “low-value care (LVC) or overuse” [5, 6]. Efforts such as Choosing Wisely [7], launched in 2012, have attempted to raise awareness regarding LVC across 500 physician-identified procedures that provide little to no patient benefit. However, these efforts do not provide a means of measuring low value care in a system.

Based on these lists, researchers have developed tools for examining LVC in large-scale claims data. For example, Schwartz et al. (2014) found that 25 to 42% of elderly Medicare beneficiaries received at least 1 of 26 low-value services in 2009, resulting in $1.9 billion to $8.5 billion in expenditures [8]. Segal, et al. (2014) produced a list of 20 “indicator procedures” representative of broader overuse within a large healthcare system [9]. This list was later applied to the Military Health System (MHS) by Koehlmoos, et al. (2019), who demonstrated the existence of LVC for the first time in the MHS among the adult beneficiaries [10]. Similarly, Chua et al. (2016) developed a novel set of 20 claim-based measures for measuring overuse in the pediatric population [11]. The study found that at least 1 in 10 commercially insured US children received one or more low-value pediatric services during 2014, resulting in $27.0 million in unnecessary spending, of which $9.2 million was paid out-of-pocket (33.9%).

The MHS provides care through a bifurcated system in which beneficiaries may receive Direct Care (DC) from providers at military treatment facilities (MTFs) including hospitals, major medical centers, and clinics, in the US and overseas; or Purchased Care (PC) through regional, non-exclusive civilian providers who accept the TRICARE insurance benefit. Building on the previous work, this study aimed to determine the degree of overuse for 20 previously published measures within the pediatric population of the MHS, as well as compare DC and PC systems for use of LVC. Results are expected to inform discussion of current plans to restructure the MHS, particularly the proposed shift of care for non-active duty beneficiaries including children to the civilian purchased care sector [12].

## Methods

### Data source and study design

We conducted a cross-sectional analysis of the 2015 Military Health System Data Repository (MDR) claims database. The MDR captures both encounter and claims data of care delivered at Military Treatment Facilities (MTFs/direct care) and at civilian fee-for-service facilities (purchased care) covered by TRICARE. TRICARE is the MHS insurance product, which provides universal coverage to approximately 9.4 million members. TRICARE beneficiaries include 20% active-duty military personnel and 80% retirees or family members, and are likely representative of the U.S. population under age 65 [13–16]. TRICARE does not include care delivered in combat zones, or through Veterans Affairs (VA) hospitals which are part of a separately administered system. TRICARE data have previously been employed in several studies designed to evaluate the quality of healthcare delivery in a variety of clinical contexts including surgical care, women’s health and pediatrics [17–19].

### Study population

Utilizing the Defense Enrollment Eligibility Reporting System (DEERS), this study identified 1,111,534 children and adolescents aged 0 to 18 years old, who were dependents of active duty and retired service members, and dependent survivors; and enrolled in TRICARE Prime benefits for the full 2015 fiscal year. As the MHS follows the Federal fiscal year calendar, the selection of dates for analysis includes October 1, 2014-September 30, 2015, and does not include the ICD-10-CM transition in October 2015. Selecting children who were dependents of active duty and retired service members, and dependent survivors ensured the study population would consistently utilize the MHS, and also eliminated the possibility of the older population (those aged 17 and 18) having enlisted status as either active duty or reservists and National Guard service members.

### Construction of low-value service measures

We adopted all 20 LVC measures in pediatric services developed by Chua et al. (2016) and which are reported here exactly as previously described [11]. No license was required in order to use this tool. Eleven of these measures were tests (6 diagnostic and 5 imaging), and the remainder were pharmaceuticals [11, 20]. Pharmaceutical codes for this study were adapted from the Market Scan therapeutic codes as used by Chua et al., and assessed in the MDR according to their American Hospital Formulary Service (AHFS) identification, as shown in Additional file 1. Because many measures reported by Chua, et al. (2016) excluded children with specific diagnosis or procedure codes in previous claims, we used the fiscal year 2014 MDR records as a “look-back period” for the identification of LVC measures in fiscal year 2015. Due to the unavailability of exact birth date in the datasets, this study was not able to exclude infants aged < 90 days or neonates aged <= 28 days, and reduced the exclusion criteria to include all infants aged 0 years. This will only influence Measure 3, Testing for Respiratory Syncytial Virus (RSV) in Children with Bronchiolitis, and Measure 8, Ultrasound in Children with Cryptorchidism. We used the narrow, more specific versions of the measures developed by Chua et al. (2016) [11].

### Statistical analyses

We calculated the proportion of children eligible for the measure who received the service at least once during fiscal year 2015, the number of services received per 100 eligible children, the proportion of children in the overall sample who received the service at least once during fiscal year 2015, and the proportion of children in the overall sample who received at least 1 of the 20 low value services during fiscal year 2015. In order to compare LVC measures in direct care vs. purchased care, patients were identified by the following criteria: 1) patients were assigned to direct or purchased care based on whether their primary care manager was in direct or purchased care, 2) those without a primary care manager were excluded, and 3) those who received any care in both direct and purchased care during fiscal year 2015 were excluded. Among children eligible for each measure, logistic regressions, adjusted by age and gender, were performed to compare the likelihood LVC in direct vs. purchased care. Due to the measure constructs and pharmacy data elements, we were unable to make comparisons in two measures (12: Cough and cold medications in children under 6 years, and 20: Acid blockers for infants with uncomplicated gastroesophageal reflux). All analyses were performed using SAS version 9.4 (SAS Institute, Inc., Cary, NC). The work was found exempt by the Institutional Review Board of the Uniformed Services University of the Health Sciences.

## Results

### Overuse procedures

Our study identified 1,111,534 eligible children (Table 1). Slightly more than 50% were male and 49% were female. The children were almost evenly split between the three age categories (0–5 year, 6–12 years, and 13–18 years) at 32.7, 37, and 30.3% respectively. In terms of primary care network, 68% of children were registered to the Direct Care setting and 31.5% were registered to receive care at civilian clinics and facilities in the Purchased Care setting.

**Table 1:** Study Population Demographics, *n* = 1,111,534

	Count (%)
**Gender**	
Male	567,086 (51.0)
Female	544,448 (49.0)
**Age Group**	
0–5	363,376 (32.7)
6–12	411,263 (37.0)
13–18	336,895 (30.3)
**Primary Care Manager’s Network**	
Direct Care	755,741 (68.0)
Purchased Care	350,625 (31.5)
None	5168 (0.5)

All 20 measures of low value care were found in the MDR (Table 2). As seen in Table 2, the number of children receiving each test, procedure, or treatment varied from 39 for neuroimaging in simple febrile seizures, to 108,574 for cough and cold medications in children under 6 years. Per 100 children in denominator, the lowest rate of use was sinus imaging for children with acute sinusitis (0.19) and the greatest rate of use was oral antibiotics for acute otitis media with effusion (41.32). Overall, 15.41% of children in our study population received at least one indicator of low-value care by the narrow measure constructs, and a total of 1,106,366 children from our study population were the eligible for the comparison analysis of direct (*n* = 755,741) vs. purchased care (*n* = 350,625).

**Table 2:** Use of 20 Low Value Service Measures and Risk Ratios

No.	Service^a^	Denominator Definition^a^	No. of Children in Denominator	No. Receiving Service at Least Once During FY	% of Denom Receiving Service at Least Once During FY	% of All Children (*n* = 1,111,543) Receiving Service at Least Once During FY	No. of Services During FY	No. of Services per 100 Children in Denominator
1	Population-based screening for vitamin D deficiency	All children	1,111,534	10,242	0.92	0.92	10,753	0.97
2	Skin prick test or IgE blood tests in children with atopic dermatitis	Children with a diagnosis of atopic dermatitis during the FY	27,659	340	1.23	0.03	356	1.29
3	Testing for RSV in children with bronchiolitis	Children with diagnosis of bronchiolitis during FY	13,813	1096	7.93	0.10	1143	8.27
4	Blood tests in children with a simple febrile seizure	Children with diagnosis of simple febrile seizure during FY	1987	129	6.49	0.01	137	6.89
5	Cervical cancer screening w/ HPV test or papanicolaou test in children	Female children aged ≥14 years	139,425	1115	0.80	0.10	1137	0.82
6	Testing for group A streptococcal pharyngitis in children aged < 3 years	Children aged < 3 years	177,669	19,467	10.96	1.75	24,979	14.06
7	Face or nose radiograph in children with head or face trauma	Children with diagnosis of head or face trauma during FY	57,943	1013	1.75	0.09	1023	1.77
8	Ultrasound in children with cryptorchidism	Children with diagnosis of cryptorchidism during FY	1823	348	19.09	0.03	354	19.42
9	Sinus imaging in children with acute sinusitis	Children with diagnosis of acute sinusitis during FY	44,399	68	0.15	0.01	85	0.19
10	Neuroimaging in children with a simple febrile seizure	Children with a diagnosis of simple febrile seizure during the FY	1987	39	1.96	0.00	39	1.96
11	Neuroimaging in children with headache	Children aged ≥12 years with a diagnosis of headache during the FY	29,801	2807	9.42	0.25	3022	10.14
12	Cough and cold medications in children aged < 6 y	Children aged < 6 years	363,376	108,574	29.88	9.77	136,381	37.53
13	Oral antibiotics for acute upper respiratory infections	Children with a diagnosis of acute upper respiratory infection during the FY	224,222	28,156	12.56	2.53	29,440	13.13
14	Oral antibiotics for acute OME	Children with a diagnosis of acute OME during the FY	26,313	10,553	40.11	0.95	10,872	41.32
15	Oral antibiotics for acute otitis externa	Children with a diagnosis of acute otitis externa during the FY	22,399	2184	9.75	0.20	2249	10.04
16	Oral antibiotics after tonsillectomy	Children undergoing tonsillectomy during the FY	6727	612	9.10	0.06	612	9.10
17	Oral antibiotics for bronchiolitis	Children with a diagnosis of bronchiolitis during the FY	13,813	2245	16.25	0.20	2368	17.14
18	Oral corticosteroids for bronchiolitis	Children with a diagnosis of bronchiolitis during the FY	13,813	2857	20.68	0.26	3222	23.33
19	Short-acting β-agonists for bronchiolitis	Children with a diagnosis of bronchiolitis during the FY	13,813	5209	37.71	0.47	5634	40.79
20	Acid blockers for infants with uncomplicated gastroesophageal reflux	Infants aged < 1 year	54,023	2395	4.43	0.22	5168	9.57

^a^Measures and denominator definitions as described by Chua, et al [11]

This study also compared results from DC vs PC (Table 3). Six services were significantly more likely in direct care, with the greatest differences observed in blood tests in children with simple febrile seizure (AOR = 24.44, 95% CI = 5.49–108.82) and testing for respiratory syncytial virus (RSV) in children with bronchiolitis (AOR = 21.01, 95% CI = 12.23–36.10). The service with the lowest odds of occurrence in direct care was cervical cancer screening with HPV test, or Papanicolau test (Pap smear) in children (AOR = 0.07, 95% CI = 0.05–0.09). Two services showed no statistical difference: ultrasound in children with cryptorchidism (AOR = 0.68, 95% CI = 0.44–1.06) and neuroimaging in children with simple febrile seizure (AOR = 1.42, 95% CI = 0.37–5.42). All measures of inappropriate antibiotic prescribing practices (measures 13 to 17) were least likely to occur in direct care, with the lowest odds observed in oral antibiotics for tonsillectomy (AOR = 0.14, 95% CI = 0.08–0.25).

**Table 3:** Comparison of Low-Value Care in Direct versus Purchased Care

No.	Service^a^	No. Receiving Service in DC	No. Receiving Service in PC	No. in DC Denominator	No. in PC Denominator	% of DC in the Denominator Receiving Service	% of PC in the Denominator Receiving Service	Adjusted^b^ OR (95% CI) for Direct Care
1	Population-based screening for vitamin D deficiency	1605	3189	386,450	306,462	0.42	1.04	0.48 (0.45–0.51)*
2	Skin prick test or IgE blood tests in children with atopic dermatitis	88	29	136	6975	64.71	0.42	3.23 (2.10–4.97)*
3	Testing for RSV in children with bronchiolitis	331	25	1876	2998	17.64	0.83	21.60 (14.29–32.65)*
4	Blood tests in children with a simple febrile seizure	22	2	180	326	12.22	0.61	24.44 (5.49–108.82)*
5	Cervical cancer screening w/ HPV test or Papanicolaou test in children	39	721	39,269	48,531	0.10	1.49	0.07 (0.05–0.09)*
6	Testing for group A streptococcal pharyngitis in children aged < 3 years	3772	4241	65,830	29,065	5.73	14.59	0.36 (0.34–0.38)*
7	Face or nose radiograph in children with head or face trauma	207	219	8892	13,910	2.33	1.57	1.98 (1.62–2.42)*
8	Ultrasound in children with cryptorchidism	45	63	282	310	15.96	20.32	0.68 (0.44–1.06)
9	Sinus imaging in children with acute sinusitis	12	40	3480	20,976	0.34	0.19	1.99 (1.03–3.81)*
10	Neuroimaging in children with a simple febrile seizure	4	5	180	326	2.22	1.53	1.42 (0.37–5.42)
11	Neuroimaging in children with headache	175	997	3084	10,632	5.67	9.38	0.59 (0.50–0.69)*
12	Cough and cold medications in children aged < 6 y	–	–	–	–	–	–	–
13	Oral antibiotics for acute upper respiratory infections	3235	9830	50,148	54,391	6.45	18.07	0.35 (0.34–0.37)*
14	Oral antibiotics for acute OME	1219	3475	3300	8419	36.94	41.28	0.82 (0.75–0.89)*
15	Oral antibiotics for acute otitis externa	333	589	4069	6092	8.18	9.67	0.82 (0.72–0.95)*
16	Oral antibiotics after tonsillectomy	13	211	677	1815	1.92	11.63	0.14 (0.08–0.25)*
17	Oral antibiotics for bronchiolitis	186	648	1876	2998	9.91	21.61	0.48 (0.40–0.58)*
18	Oral corticosteroids for bronchiolitis	276	684	1876	2998	14.71	22.82	0.62 (0.53–0.72)*
19	Short-acting β-agonists for bronchiolitis	795	1158	1876	2998	42.38	38.63	1.16 (1.03–1.31)*
20	Acid blockers for infants with uncomplicated gastroesophageal reflux	–	–	–	–	–	–	–

^a^Measures and denominator definitions as described by Chua, et al [11]

^b^Logistic regression models for each measure were adjusted by age and gender

*Statistically significant Adjusted Odds Ratio (AOR) and 95% Confidence Interval (CI), *p*-value < 0.05

– Data unavailable due to inability to compare direct vs purchased care under defined measure constructs

## Discussion

In this study, 1 in 7 MHS children received at least one of 20 low-value services during fiscal year 2015. Rates of low-value service use differed according to whether care was delivered in direct versus purchased care, with 6 services significantly more likely to be delivered in direct care and 10 services significantly more likely to be delivered in purchased care. A greater difference in antibiotic prescription practices was also observed, with all 5 services more likely to be received in purchased versus direct care.

The MHS, due to its bifurcated structure, enables a comparison of the provision of care under different payment models, and is especially relevant to discussions of the MHS transition to a high-reliability organization focused on value-based care. Simultaneously, the MHS is planning to reduce the numbers of its uniformed providers, including those providing pediatric care, by 17,000 positions in order to fulfill priorities focused on military readiness [12]. Such changes would shift the vast majority of pediatric services for TRICARE beneficiaries to purchased care. In this context, the discussion of low-value pediatric care is both timely and relevant, as it provides an opportunity to reduce costly care that provides no benefit, reduce harm to patients, and repurpose existing resources for more efficient and appropriate use of care. Therefore, this study informs discussion by identifying opportunities to improve pediatric care across the MHS, and evaluating differences in low-value pediatric care between the DC and PC environments.

We found that six low-value procedures are significantly more likely to be provided in direct care: skin prick or IgE blood test for atopic dermatitis, testing for RSV in children with bronchiolitis, blood tests for simple febrile seizure, face or nose radiograph for children with head or face trauma, sinus imaging in children with acute sinusitis, and short-acting beta-antagonists for bronchiolitis. The prevalence of face or nose radiographs after trauma may reflect current military focus on preventing traumatic brain injury, which originates from practice management implications in the adult population. One set of procedures, performing HPV test or Pap smear on children, was much less likely to be performed in direct vs. purchased care. The lower rate in direct care may be the result of outsourcing of care to civilian facilities rather than an overt focus on providing fewer of these procedures across the MHS.

Further insight is gained from comparing rates in the MHS to those seen in private insurance systems. Chua, et al. [11] reported that 9.6% of children received at least one low-value procedure during 2014. In contrast, 15.4% of children in the MHS received at least one low-value procedure during 2015. A follow-up study by Chua, et al. [21] found 1 in 9 publicly insured and 1 in 11 privately insured children receiving LVC in 2014, concurring with earlier findings and demonstrating relatively little difference between their two payment systems.

Significant differences in the rates of two services involving the prescription of oral antibiotics were observed in the MHS compared to publicly and privately insured children. In this study, 9.8% of the eligible population of military children received oral antibiotics for acute otitis externa, and 9.1% received oral antibiotics after tonsillectomy. These rates are significantly lower than those found by Chua, et al. (2020) in publicly and privately insured children, with 25.3% of publicly insured and 24.3% of privately insured children receiving oral antibiotics for acute otitis externa, and 29.0% of publicly insured and 27.5% of privately insured children receiving oral antibiotics after tonsillectomy.

Another notable difference was found in the prescription of cough and cold medicines. In this study, 30% of the eligible population of military children received one or more cough or cold medicines during the study period, in contrast to the previously-reported 8.5% of publicly insured and 3.2% of privately insured children [11, 20]. A review of coding indicates that this is a true difference and not due to different definitions of these medications between studies. Although the reason for this difference cannot be determined from the data set, it may be due to the availability of these medications in the MHS formulary, in which they can be obtained at no cost to the patient when obtained at a Military Treatment Facility or at a very low co-payment at a networked pharmacy, vs. other systems in which the patient bears some cost in obtaining them from a pharmacy or as an over-the-counter product.

A notable finding is the difference in antibiotic prescription patterns between purchased and direct care. In all five cases, children in the direct care system were less likely to receive low-value antibiotic prescriptions (oral antibiotics for acute upper respiratory infection, for otitis media with effusion, for otitis externa, for bronchiolitis, and after tonsillectomy) than in the purchased care system. This is a particular concern in light of current discussions of antibiotic-resistant disease driven in part by inappropriate prescribing practices, although data did not illustrate the reason for the difference between the two care systems.

### Strengths and limitations

There are several notable strengths in this investigation. The population is comprised of a large number of children and adolescents (*n* = 1,111,534) who receive care through universal insurance, and as such, may provide a model for practice patterns in the setting of insurance expansion in the greater US population. Additionally, the provision of care through different practice environment presents opportunities to examine the effect of fixed-fee (direct care) or fee-for-service (purchased care) payment models on the delivery of low-value care. Notably for this study, it appears that low-value pediatric care is provided with variation between the fixed-fee vs. fee-for-service system, with greater use of low-value antibiotic treatment taking place in the purchased care sector. This is in similar to previous studies in the MHS adult population which showed utilization of less-invasive procedures, and greater adherence to treatment guidelines, in direct care [10, 16, 21, 22]. Our current findings suggest that current plans to shift pediatric health services in the MHS to purchased care may result in an increase in the use of some low value services while increasing the inappropriate use of antibiotics.

We also recognize several limitations in this work. First, the use of administrative data renders it susceptible to any errors in coding at the provider level, and does not capture the nuances of care in which use of low value procedures may be clinically appropriate. Second, given the use of de-identified data, this study does not have full date of birth for its patients, so that tests provided to children in the first year of life may not be accurately captured.

## Conclusions

Using 20 previously developed measures, this study found evidence of low-value care in pediatric health services in the MHS. Significant differences existed between the provision of low value services in direct and purchased care settings, as well as between the MHS and previous research relying on patients with private insurance. Further research is needed to understand the etiologies behind these differences. In the current period of MHS reform, as pediatric health services are increasingly shifted to the purchased care, the results of our work indicate that such policies may result in increases in low value care and associated expenditures including an increased risk of inappropriate antibiotic use.

## Supplementary information

**Additional file 1.** Drugs Used in Measure Definitions. Names and codes of drugs used for investigation of Measures 13–20. Separately uploaded in accordance with Journal guidelines.

## Data Availability

The data that support the findings of this study are available from the United States Defense Health Agency. Restrictions apply to the availability of these data, which were used under federal Data User Agreements for the current study, and so are not publicly available.

## Abbreviations

AHFS: American Hospital Formulary Service
AOR: Adjusted Odds Ratio
CI: Confidence Interval
DC: Direct Care
DEERS: Defense Enrollment Eligibility Reporting System
Denom: Denominator
DoD: Department of Defense
HPV: Human Papillomavirus
LVC: Low Value Care
MDR: Military Health System Data Repository
MHS: Military Health System
NA: Not Applicable
Num: Numerator
PC: Purchased Care
RSV: Respiratory Syncytial Virus
US: United States
VA: Veterans Affairs
