# Supplementary material for: Use of low-value pediatric services in the Military Health System
Source: BMC Health Serv Res. 2020 Aug 20;20:770. doi: 10.1186/s12913-020-05640-5 (PMC7441552; doi:10.1186/s12913-020-05640-5)
Supplement: Supplementary file 1 — Additional file 1. Drugs Used in Measure Definitions. Names and codes of drugs used for investigation of Measures 13–20. Separately uploaded in accordance with Journal guidelines. [file 12913_2020_5640_MOESM1_ESM.docx]

Additional File 1: Drugs Used in Measure Definitions

| **Measure** | **American Hospital Formulary Service (AHFS) Therapeutic Classes or Prescription Drug Names Used in Measure Definition** |
| --- | --- |
| Measure 13: Oral Antibiotics for Upper Respiratory Tract Infections | 081206, 081207, 081212, 081216, 081224, 081200 (cephalosporins, B-lactam antibiotics, macrolides, penicillins, tetracyclines, antibiotics (systemic)) |
| Measure 14: Oral Antibiotics for Acute Otitis Media with Effusion | 081206, 081207, 081212, 081216, 081224, 081200 (cephalosporins, B-lactam antibiotics, macrolides, penicillins, tetracyclines, antibiotics (systemic)) |
| Measure 15: Oral Antibiotics for Acute Otitis Externa | 081206, 081207, 081212, 081216, 081224, 081200 (cephalosporins, B-lactam antibiotics, macrolides, penicillins, tetracyclines, antibiotics (systemic)) |
| Measure 16: Oral Antibiotics after Tonsillectomy | 081206, 081207, 081212, 081216, 081224, 081200 (cephalosporins, β-lactam antibiotics, macrolides, penicillins, tetracyclines, antibiotics (systemic)) |
| Measure 17: Oral Antibiotics for Bronchiolitis | 081206, 081207, 081212, 081216, 081224, 081200 (cephalosporins, B-lactam antibiotics, macrolides, penicillins, tetracyclines, antibiotics (systemic)) |
| Measure 18: Oral Steroids for Bronchiolitis | All oral forms of prednisone/prednisolone: 481008 (corticosteroids) |
| Measure 19: Short-acting β-agonists for Bronchiolitis | Generic and brand name short-acting β-agonists containing: Albuterol, Pirbuterol Levalbuterol, Metaproterenol |
| Measure 20: Reflux Medications for Infants | Histamine_2_-blocker or proton pump inhibitor, excluding aripiprazole: 562812, 562836 |
